# The attitudes of mental health professionals on the employability of people with mental illness: A different view limiting employment rehabilitation

**DOI:** 10.1002/brb3.2767

**Published:** 2022-09-13

**Authors:** Andrea Lettieri, Felipe Soto‐Pérez, Emiliano Díez, Mara Bernate‐Navarro, Manuel Franco‐Martín

**Affiliations:** ^1^ Faculty of Psychology University of Salamanca Salamanca Spain; ^2^ INTRAS Foundation Zamora Spain; ^3^ INICO: University Institute for Community Integration Salamanca Spain; ^4^ IBSAL: Institute of Biomedical Research of Salamanca Salamanca Spain; ^5^ Department of Psychiatry Hospital Provincial Rodríguez Chamorro Zamora Spain

**Keywords:** attitudes of health personnel, employment, people with disability, surveys and questionnaires

## Abstract

**Objectives:**

Mental health professionals are becoming increasingly involved in the process of employment rehabilitation of persons with psychiatric disabilities. However, few studies address the attitudes of these professionals toward the employability of those with mental illness. The aim of this research was to identify differences in the attitudes of medical and non‐medical mental health professionals, as well as to detect any association between attitude scores and the type of professional.

**Methods:**

A sample of 140 employees from public and third sector mental health organizations answered a questionnaire using a scale measuring their attitudes and views on the employability of people with psychiatric disabilities. The psychometric characteristics of the scale are provided together with the variations detected in the professionals’ attitudes.

**Results:**

This research shows that significant differences in the attitudes between medical and non‐medical mental health employees exist and that there is a need for the implementation of educational programs that may help to improve the attitudes of medical professionals toward the employability of people with mental illness.

**Conclusion:**

This research indicates the importance of improving the professionals’ attitudes to support people attempting to return to work.

## 1 INTRODUCTION

Disability is the result of the interaction between the health condition of an individual and the environmental factors that together determine the person's functionality, activity, and level of participation in society (World Health Organization [WHO], 2001). Paid work is the main form of participation in which people can feel useful and productive (Koletsi et al., 2009; Ryan et al., 2012; Saavedra et al., 2016; Torres Stone et al., 2018). However, despite the efforts made over the last few decades, people with a disability still have fewer job opportunities than people without a disability. Moreover, people with mental illness are even less likely to find and maintain a paying job (WHO, 2000; Organisation for Economic Co‐operation and Development [OECD], 2012, 2018), as there are many barriers that limit their access to employment (Foster et al., 2019; Lettieri & Díez, 2017). Aside from the intrinsic characteristics related to the correct functioning of this group of people (McGurk & Mueser, 2013), different studies show there are many other environmental factors involved that may support or limit work inclusion (Harris et al., 2014; Hong et al., 2014; Lettieri & Díez, 2017; Moody et al., 2017; Netto et al., 2016). Many of these studies focus on the quality of training and supported employment programs that were developed in the United States and then exported and tested in other countries worldwide (Gewurtz et al., 2012; Grove, 2015; Koletsi et al., 2009; Suijkerbuijk et al., 2017; Waghorn et al., 2017, 2020). The Individual Placement and Support (IPS) is a supported employment model that showed evidence of efficacy for gaining competitive employment, compared with traditional work rehabilitation models (Kinoshita et al., 2013; Suijkerbuijk et al., 2017). The IPS is focused on obtaining competitive employment for people with mental illness through a rapid job search and implementing close integration between employment and mental health teams (Bond et al., 2020). However, the mental health professionals’ expectations about the employability of people with mental illness may limit the implementation of IPS or other supported employment programs and with possible negative effects on the beliefs of employers and people with mental illness (Rinaldi et al., 2008).

Even though interest in this research area is focused on improving these types of programs and although stigma and attitudes toward mental illness have been analyzed in great depth (Couture & Penn, 2003; Lagunes‐Cordoba et al., 2020; Maunder & White, 2019; Morgan et al., 2016), as well as the self‐stigma process (Corrigan et al., 2012; Rüsch et al., 2014), not much research has been conducted on addressing how the stigma of mental illness can limit people with psychiatric disabilities in obtaining a competitive job (Dolce & Bates, 2019; Jansson & Gunnarsson, 2018; Lettieri & Díez, 2017; Lettieri, Díez, et al., 2021; Ottewell, 2019).

The employers’ opinions, and those of family, friends, and medical and non‐medical mental health professionals, can be crucial for both returning to work and work inclusion of people with psychiatric disabilities (Lettieri et al., 2022). Research on the attitudes of mental health professionals toward the employability of people with mental illness, as well as the stakeholders involved in promoting and supporting employment for these people, provide important insight for addressing this issue. Nonetheless, while there are reports that detail the points of view of employers (Hand & Tryssenaar, 2006; Jansson & Gunnarsson, 2018; Lettieri, Díez, et al., 2021; Mangili et al., 2011; Ozawa & Yaeda, 2007; Unger, 2002), there are not that many focusing on the attitudes and perceptions of mental health professionals toward the employability of people with mental illness (Fleming et al., 2019; Gladman et al., 2015; Lettieri, Soto‐Pérez, et al., 2021). The role of these professionals is crucial for helping people not only medically but also to help them achieve a better quality of life through the positive experience of obtaining a job and their subsequent recovery (Bertilsson et al., 2015; Gladman et al., 2015; Porter, et al., 2018). Also, it is recognized that in order to achieve a suitable implementation of the IPS‐supported employment program, there must be a common goal (or at least good coordination) between job coaches and mental health psychiatrists, psychologists, and nurses (Becker & Drake, 1994; Hillborg et al., 2013). Thus, having similar beliefs about the capability of people with mental illness and their employability may help to create greater team cooperation and obtain better employment outcomes.

Some research on attitudes toward mental illness has revealed that despite professionals being more knowledgeable about mental health, they can still form stereotypes and social distance (Nordt et al., 2006). Also, when considering different types of professionals, psychiatrists seem to have attitudes similar to those of other mental health medical staff. By contrast, other therapists, like psychologists or social workers, seem to have more positive attitudes toward mental illness (Nordt et al., 2006; Olmo‐Romero et al., 2019). Non‐medical professional groups pertaining to non‐governmental organizations (NGOs) also show more positive attitudes when compared with other medical mental health professionals (Lettieri, Soto‐Pérez, et al., 2021; Rose et al., 2018). Despite general opinions about the importance of employment for recovering a meaningful life (Gladman et al., 2015), a recent study suggests that medical mental health professionals consider people with mental illness to be less capable of carrying out full‐time jobs in 23% of the cases, 45% in part‐time employment, and the rest being incapable of any type of remunerated work (Fleming et al., 2019). Similarly, another recent study showed that only half of professionals strongly agree that people with mental illness want work and, most importantly, around the same proportion moderately agree that people with mental illness cannot work (Brucker & Doty, 2019).

The medical mental health professionals seem to have some doubt about the positive effect of work on recovery (Casper & Carloni, 2007), believing that work may be a source of stress that may worsen mental illness symptoms, and so people must wait to be clinically stable before seeking a job (Hamilton et al., 2013; Marwaha et al., 2009; Mueser & McGurk, 2014). This issue has great importance because medical mental health professionals have an important counseling role in supporting people with mental illness recovery, and there are studies that show how the opinions of these staff may discourage people from working (Costa et al., 2017; Noel et al., 2017; Roets et al., 2007). The negative expectations and fears about the worsening of mental health because of work is an important barrier reported by people with psychiatric disabilities too, and mental health professionals may have a negative effect confirming and sharing this belief (Lettieri et al., 2022).

All this research seems to reinforce the theory that if a professional only provides medical support, they remain unaware of how the person is performing on the job when they are going through a positive phase. As a result, the expectations of the medical professionals regarding the employment prospects of people with mental illness are not positively influenced (Cohen & Cohen, 1984; Fleming et al., 2019; Nordt et al., 2006; Olmo‐Romero et al., 2019; Rose et al., 2018).

After examining the information appearing in the literature regarding this topic, we believe further research is necessary for obtaining more in‐depth knowledge about this relationship. For this reason, the purpose of the present study is to explore the opinions of medical and non‐medical mental health professionals on the employability of people with mental illness. Non‐medical professionals are more aware of the daily activities and the performance of people with mental illness in their jobs than medical professionals, who are in greater direct contact with patients when they are suffering from an acute psychiatric episode or for treatment adherence in a hospital. Thus, the hypothesis for this study is that the opinions on the employability of people with mental illness will be significantly worse in the case of medical professionals, compared with non‐medical professionals of mental health.

## 2 METHOD

### 2.1 Participants

The questionnaire was applied to a sample of 140 professionals from mental health organizations mainly located in the Spanish provinces of Zamora (35%), Valencia (19.3%), Valladolid (17.9%), and Madrid (13.6%), with the rest being located in Bizkaia, Caceres, and Zaragoza (14.3%). The age of the participants ranged from 21 to 62 years (*M* = 37.3, standard deviation [*SD*] = 10.04) and the majority of the participants were women (71.4%). The educational levels were the following: high school graduates or intermediate‐level vocational training (5%), higher‐level vocational training (7.1%), university education (43.6%), and a master's degree, medical specialization, or a PhD (44.3%). The sample comprised non‐medical professionals employed by NGOs (62.9%) and by medical professionals working in mental health hospitals (37.1%). The participants worked mainly in psychosocial (18.6%), psychiatric (16.4%), and psychological (14.3%) support roles, followed by rehabilitation work (10%), housing support (8.6%), nursing care (10%), and leisure and free time (7.1%). The remaining participants worked at services not directly associated with health assistance such as administrative work and human resources (15%).

### 2.2 Sampling procedure

All participants were invited to take part in the survey via email, consisting of a message and the link to the survey form developed through the Qualtrics platform. For non‐medical professionals associated with NGOs, the inclusion criteria stipulated that the organizations should mainly provide services for people with psychiatric disabilities, have at least 10 years of activity, and, at the time of the survey, be providing assistance to 500 individuals in more than one city. The procedure for searching for NGOs was done using the Internet. Finally, eight mental health third sector NGOs were contacted, each one from a different region in Spain. Of these, four agreed to participate in the study and sent an email to their employees inviting them to participate in a research study. Two organizations refused to take part in the study because they had several surveys underway at the time of this proposal, and two organizations did not respond to the invitation email. The medical‐related mental health professionals participating in the study were selected from short training courses organized in 2016, 2018, and 2019 by Janssen and the mental health hospital in Zamora (Acta Sanitaria, 2016). The inclusion criterion for these participants was that they worked as psychiatrists, nurses, or psychologists in a mental health hospital in Spain. The respondents were asked to sign the consent form, which was the first page of the survey, before carrying on with completing the questionnaire. Data were collected between March 2016 and December 2019, and all information from participants not completing the survey was deleted.

### 2.3 Instrument

For this study, we used an instrument recently developed in Spain to measure the attitudes toward the employability of people with mental illness (CEPEM: Questionnaire about the employability of people with mental disorders). This scale was previously used with employers and mental health professionals (Lettieri, Soto‐Pérez, et al., 2021), providing the first explorative results and showing acceptable psychometric properties. The last version of the scale, after being checked for internal consistency, provided 33 items distributed into the content spheres of attitudes toward (1) people with mental illness, (2) their employability, and the (3) socio‐economic impact on the company. The second validation analysis of the usability of this scale only considered the sample of employers and showed a bi‐factor dimensionality of the instrument, with the first and second theorized dimensions merged into an attitude and impact factor (Lettieri, Díez, et al., 2021). However, to date, this scale has not been tested with factor analysis on a sample of mental health professionals. The scale uses a Likert‐type response format with scores that range from 1, *totally disagree*, to 7, *totally agree*. In the addition to ranking attitudes, the questionnaire also collected socio‐demographic data on age, gender, the level of education, as well as information about the professional sector in which the respondents were working; each individual was asked to be specific about their profession (psychiatrist, nurse, work rehabilitator, etc.). Data were also collected on the type of contacts the professionals had (family members, friends, neighbors, etc.) and the number (from none to more than five) of people with mental illness.

### 2.4 Data analysis

First, the psychometric characteristics of the scale were examined, and the results showed that the scale was appropriate for measuring the attitudes of the mental health professionals in the sample group. Then, we tested whether the factor structure of the attitude scale would be confirmed in the mental health sample. Finally, the factor score results were used to highlight the different attitudes that medical and non‐medical mental health professionals may have.

### 2.5 Psychometric analysis

The consistency analysis was computed following the evidence established in the literature. First, we reviewed the normality distribution of each item, selecting, as a consequence, the dispersion matrix and method for the exploratory factor analysis (EFA). The number of dimensions was assigned through the optimal implementation of parallel analysis (PA), and robust promin rotation was pre‐established because the factors are expected to be correlated with each other. Items with loadings greater than 0.50 were kept and the factor program was used to compute the EFA (Lorenzo‐Seva & Ferrando, 2006, 2013).

### 2.6 Model evaluation

The comparative fit index (CFI), Tucker–Lewis index (TLI), and the root mean square error of approximation (RMSEA) were used as fit indices (Bentler, 1990; Steiger, 1990; Tucker & Lewis, 1973). As established in other works (Browne & Cudeck, 1992; Hu & Bentler, 1998; Morin et al., 2016), the CFI and TLI indices are considered to be adequate or an excellent fit if they are greater than 0.90 and 0.95, respectively, while for RMSEA, the value should be lower than 0.08 and 0.06.

### 2.7 Factor tests used on mental health medical and non‐medical professionals

Using a psychometric analysis, factor results and scores were obtained for the entire sample. In this second phase, the factor scores were analyzed in association with the socio‐demographic variables gathered from mental health professionals. We explored if attitudes have significant associations with age, gender, and level of education. Then, we analyzed if there were significant differences between the attitudes of mental health medical and non‐medical professionals and among specific professions. Finally, we analyzed if having contact with people with mental illness (like relatives, friends, and neighbors) caused attitudes to differ. The *t*‐tests, ANOVA with Bonferroni post hoc and Mann–Whitney tests, and Spearman correlations were used for all of these analyses.

## 3 RESULTS

### 3.1 Psychometric properties of the scale

The SDs of the results obtained showed inacceptable variability for 10 items in the scale (*SD* < 1.2), while another six items were excluded from the analysis because of non‐significant item‐total correlation (*r* < .30) or because of their distributions (with not all categories observed in variables). The majority of these items were strictly associated with attitudes toward people with mental illness (e.g., *people who have a mental illness should not be allowed to vote*), while the rest were items mostly oriented toward registering opinions about the impact felt by companies due to hiring people with mental illness (e.g., *hiring a person with mental illness improves the company's image of social commitment*). As the Kolgomorov‐Smirnov‐tests revealed no significant normal distribution for the remaining 17 items, we proceeded to use a polychoric correlations matrix for the EFA.

Robust PA revealed one advised dimension. The factor was extracted using the robust unweighted least squares method, with correction for Chi‐square using robust mean and variance‐scaled (Asparouhov & Muthén, 2010). With EFA, eight items were progressively excluded as their factor loadings were below 0.50. The Kaiser–Meyer–Olkin (KMO) test showed the goodness of fit for the correlation matrix (KMO = 0.84, 95% CI = [0.82, 0.89]), with nine items loading adequately into a factor (work functioning factor) strictly related to opinions on how individuals with mental illness function at work and the convenience of hiring people with this disability (Table 1). Robust PA confirmed one advised dimension, excluding multidimensional solution through the unidimensional congruence value (UniCo = 0.97, 95% CI = [0.953, 0.988]) and the mean of item residual absolute loadings (MIREAL = 0.25, 95% CI = [0.200, 0.288]). Good reliability was obtained for the scale (Cronbach's alpha = .84), with robust goodness of fit statistics showing an adequate result for RMSEA (RMSEA < 0.08, 95% CI = [0.04, 0.09]), as well as for the CFI (CFI = 0.97, 95% CI = [0.96, 0.99]), the non‐normed fit index (TLI = 0.97, 95% CI = [0.94, 0.99]), and for the weighted root mean square residual (WRMR = 0.08, 95% CI = [0.06, 0.10]). Factor scores were extracted and introduced into the database for further analyses.

**TABLE 1:** Means, standard deviations (SDs) and factor loadings of selected items after exploratory factor analysis

	Mean	SD	Factor loadings
V 20. It is not convenient to hire a person with mental illness because it does not ensure work continuity due to their unstable health	5.82	1.36	0.765
V 3. People with mental illness often have problems of absenteeism and punctuality in their jobs	5.24	1.39	0.724
V 2. Many people with mental illness find it difficult to follow work instructions properly	5.06	1.53	0.664
V 32. People with mental illness do not want to work	6.02	1.37	0.654
V 5. People with mental illness experience too much frustration at work	4.82	1.50	0.638
V 19. Training a person with mental illness for a job in a company would take a greater amount of time and money than training someone without mental illness	6.04	1.24	0.631
V 27. Many people with mental illness are too slow to work efficiently	5.41	1.51	0.628
V 6. The productivity of people with mental illness can be as good as that of people without disabilities	5.89	1.39	0.615
V 8. If a person with mental illness does not work for years, it will be more difficult to return to work and be efficient and productive	4.19	1.82	0.577

*Note*. Only the mean of Item “*V 6”* not represent a reverse scoring.

### 3.2 Attitude differences between medical and non‐medical mental health professionals

This section presents the results obtained for sample differences tests on the factor score extracted from the attitude scale. These analyses show the associations among the socio‐demographic variables as well as differing attitudes among the type of professionals. Moreover, some differences in attitude associated with variables regarding contact are also shown.

First, our results showed no significant differences in the scores related to gender (men, *M* = −0.05, *SD* = 0.73; women, *M* = 0.02, *SD* = 0.95), *t*(138) = −0.42, *p* > .05, 95% CI = [−0.40, 0.26], *r* = .04. With respect to educational level, a recoded variable was created using five groups of participants (medium vocational training, high vocational training, undergraduate, masters, and PhD). No significant correlation was obtained for professionals with different educational levels, *r*_s_(138) = .01, *p* > .05. Also, while no significant differences were found for gender and education, the age variable showed a significant but small negative correlation with the factor score, *r*_s_(138) = −0.25, *p* < .01.

The main objective of this study was to examine differences in attitudes between medical and non‐medical mental health professionals. First, we focused on relevant factor score differences between groups. Then, we analyzed the content of specific items that seemed to determine the more relevant differences.

First, the results of the *t*‐test indicated that the mental health medical professionals had a significant lower score (*M* = −0.33, *SD* = 0.95) than the non‐medical professionals (*M* = 0.20, *SD* = 0.80), *t*(138) = −3.49, *p* < .001, 95% CI = [−0.82, −0.23], *r* = .28, *d* = −.59. Second, an analysis of variance revealed significant differences in the factor score based on the type of profession: *F*(7,132) = 6.13, *MSE* = 0.64 *p* < .001, $\eta_{p}^{2}$ = 0.25, with the Bonferroni post hoc test revealing a significant lower result for psychiatrists (*M* = −0.57, *SD* = 0.74), compared with psychologists (*M* = 0.50, *SD* = 0.61), (*p* < .001, 95% CI = [−1.84, −0.29], *d* = −1.19). This was also the case when comparing psychiatrists with people working in the home and community support services (*M* = 0.37, *SD* = 0.93), (*p* < .05, 95% CI = [−1.84, −0.03], *d* = −1.05) and for people working in psycho‐social rehabilitation services (*M* = 0.41, *SD* = 0.56), (*p* < .001, 95% CI = [−1.71, −0.25], *d* = −1.10). The same set of comparisons also showed significant lower factor scores for nurses (*M* = −0.72, *SD* = 1.11) when compared with psychologists, (*p* < .001, 95% CI = [−2.10, −0.33], *d* = −1.36), as well as with home and community support professionals, (*p* < 0.05, 95% CI = [−2.09, −0.09], *d* = −1.21), and with psycho‐social rehabilitators, (*p* < .001, 95% CI = [−1.97, −0.29], *d* = −1.26). Considering that psychologists had the highest average factor score when comparing the different professional groups, we proceeded to analyse if this result was different for psychologists working in hospitals or third sector non‐medical organisations. The result of the *t*‐test showed there were no significant differences between psychologists working in medical (*M* = 0.80, *SD* = 0.70) and non‐medical contexts (*M* = 0.30, *SD* = 0.67), *t*(18) = 1.64, *p* = .12, 95% CI = [−0.14, 1.14]. This suggested that psychologists have a better opinion on the employability of people with psychiatric disabilities irrespective of if they are working in hospitals or not.

Mann–Whitney tests were then performed to detect any significant differences in the item scores between medical and non‐medical mental health professionals, as well as specific professions. As can be seen in Table 2, medical mental health professionals have a worse opinion of the employability of people with mental illness than non‐medical employees. Specifically, the medical professionals had worse opinions about the difficulty that people with mental illness have to adequately follow instructions and work quickly. They seemed to consider people with mental illness as being less productive because they have less time active in the workforce, have more problems with absenteeism and arriving late, and because of the feeling of frustration when dealing with job tasks that present certain difficulties (Table 2). The sample comparisons between professions revealed that both psychiatrists and nurses have a significantly worse opinion than non‐medical employees like psychologists, psycho‐social rehabilitators, and home and community support professionals. However, the results revealed that each group showed significant differences with regard to specific items. For example, psychiatrists had a worse opinion than non‐medical professionals about people with mental illness as they may have problems of absenteeism or punctuality, a general difficulty in being productive because of their long work inactivity, and feelings of frustration when they are working. By contrast, nurses seemed to have a worse opinion about employability. They considered it a waste of time for companies to train people with mental illness for a job, especially considering their unstable health, their lack of persistence in carrying out the work and because they believed people with mental illness do not want to work (Table 2).

**TABLE 2:** Mann–Whitney test results for differences in items score for medical and non‐medical professionals and for significant comparisons of various professions with psychiatrists and nurses

	Med versus non‐med	Psychi versus psycho	Psychi versus PS rehab	Psychi versus home and CS	Nurses versus psycho	Nurses versus PS rehab	Nurses versus home and CS
V 2. Many people with mental illness find it difficult to follow work instructions properly	U = 1568 Z = −3.21 *D* = −0.27**	U = 51 Z = −4.49 *D* = −0.68***	U = 103 Z = −4.01 *D* = −0.57***	U = 62 Z = −2.69 *D* = −0.45**	U = 51 Z = −3.34 *D* = −0.57***	U = 89 Z = −2.73 *D* = −0.43**	U = 47 Z = −1.91
V 3. People with mental illness often have problems of absenteeism and punctuality in their jobs	U = 1803 Z = −2.178 *D* = −0.18*	U = 87 Z = −3.6 *D* = −0.55***	U = 136 Z = −3.38 *D* = −0.48***	U = 55 Z = −2.96 *D* = −0.50**	U = 78 Z = −2.27 *D* = −0.39*	U = 119 Z = −1.86	U = 50 Z = −1.82
V 5. People with mental illness experience too much frustration at work	U = 1758 Z = −2.34 *D* = −0.20*	U = 101 Z = −3.21 *D* = −0.49**	U = 166 Z = −2.71 *D* = −0.39**	U = 74 Z = −2.26 *D* = −0.38*	U = 77 Z = −2.25 *D* = −0.39*	U = 116 Z = −1.90	U = 53 Z = −1.62
V 6. The productivity of people with mental illness can be as good as that of people without disabilities	U = 1754 Z = −2.44 *D* = −0.21*	U = 159 Z = −1.79	U = 195 Z = −2.17 *D* = −0.31*	U = 74 Z = −2.30 *D* = −0.39*	U = 102 Z = −1.40	U = 121 Z = −1.82	U = 46 Z = −2.04 *D* = −0.40*
V 8. If a person with mental illness does not work for years, it will be more difficult to return to work and be efficient and productive	U = 1748 Z = −2.36 *D* = −0.20*	U = 93 Z = −3.38 *D* = −0.52***	U = 112 Z = −3.81 *D* = −0.54***	U = 51 Z = −3.06 *D* = −0.52**	U = 97 Z = −1.55	U = 121 Z = −1.77	U = 56 Z = −1.44
V 19. Training a person with mental illness for a job in a company would take a greater amount of time and money than training someone without mental illness	U = 1847 Z = −2.04 *D* = −0.17*	U = 220 Z = −0.25	U = 216 Z = −1.86	U = 97 Z = −1.57	U = 68 Z = −2.62 *D* = −0.49**	U = 56 Z = −3.84 *D* = −0.61***	U = 27 Z = −3.07 *D* = −0.60**
V 20. It is not convenient to hire a person with mental illness because it does not ensure work continuity due to their unstable health	U = 1967 Z = −1.46	U = 168 Z = −1.58	U = 202 Z = −2.05 *D* = −0.29*	U = 130 Z = −0.27	U = 80 Z = −2.20 *D* = −0.38*	U = 95 Z = −2.63 *D* = −0.42**	U = 62 Z = −1.14
V 27. Many people with mental illness are too slow to work efficiently	U = 1669 Z = −2.75 *D* = −0.23**	U = 118 Z = −2.82 *D* = −0.43**	U = 198 Z = −2.07 *D* = −0.30*	U = 73 Z = −2.32 *D* = −0.39*	U = 52 Z = −3.19 *D* = −0.55**	U = 95 Z = −2.53 *D* = −0.40*	U = 55 Z = −2.57 *D* = −0.50*
V 32. People with mental illness do not want to work	U = 1973 Z = −1.49	U = 144 Z = −2.34 *D* = −0.36*	U = 237 Z = −1.35	U = 98 Z = −1.49	U = 70 Z = −2.76 *D* = −0.47**	U = 117 Z = −1.99 *D* = −0.31*	U = 48 Z = −1.96 *D* = −0.38*

*Note*: D = Cohen effect size; med = medical, non‐med = non‐medical, psychi = psychiatrists, psycho = psychologists, PS rehab = psycho‐social rehabilitators, home and CS = home and community support.

^***^ *p* < .001;

^**^ *p* < .01;

^*^ *p* < .05.

Last, we analyzed the association between variables related to contact and factor scores. In doing so, we explored if having friends, relatives, or neighbors with mental illness influenced the opinions of mental health professionals on the employability of people with mental illness. The *t*‐tests showed that there was no significant difference in the score obtained for those who had one or more friends with this disability (*M* = −0.03, *SD* = 0.95) than those who did not (*M* = −0.07, *SD* = 0.78), *t*(138) = 0.61, *p* > .05, 95% CI = [−0.42, 0.22]. The same result was found for contact in the form of relatives with mental illness (with one family member or more, *M* = −0.08, *SD* = 1.02, or with no family members, *M* = 0.05, *SD* = 0.81), *t*(138) = −0.85, *p* > .05, 95% CI = [−0.17, 0.44]), or neighbors with this disability (with one neighbor or more, *M* = −0.03, *SD* = 0.96, or no neighbors, *M* = 0.05, *SD* = 0.80), *t*(138) = −0.51, *p* > .05, 95% CI = [−0.23, 0.38]. The same non‐significant patterns were obtained for both groups of medical and non‐medical mental health professionals.

## 4 DISCUSSION

This study is an initial step in validating the psychometric properties of an instrument measuring the attitudes of mental health professionals toward the employability of people with mental illness expanding upon what has already been reported (Fleming et al., 2019; Gladman et al., 2015; Lettieri, Soto‐Pérez, et al., 2021; Olmo‐Romero et al., 2019; Porter et al., 2019; Rose et al., 2018). In addition to showing good psychometric results, the instrument is novel in that it assesses the attitudes of mental health professionals, differentiating their views on the work performance of people with mental illness from those associated with the impact and desirability of employing people with mental illness. The result of the factor analysis suggests the possibility of further studies, extending the research toward the analysis of the process of attitude change before and after the implementation of employment programs in which mental health employees are directly involved in supporting people in their work rehabilitation.

The results of this research corroborate that medical mental health professionals have a significantly worse opinion than NGO non‐medical professionals when considering the work performance of individuals (work functioning factor). These different mindsets partially confirm previous studies (Fleming et al., 2019; Nordt et al., 2006; Olmo‐Romero et al., 2019; Rose et al., 2018), where non‐medical professionals have a better view of the employability of people with mental illness. Consequently, differing views between medical and social professionals may reduce the effectiveness of work rehabilitation programs, discouraging or confusing individuals who need to increase their intrinsic and extrinsic motivation to work (Reddy et al., 2016), which is a requirement for individuals to develop an active and action‐oriented attitude toward employment (Brantschen et al., 2017). This research also confirms other previous studies (Brucker & Doty, 2019; Fleming et al., 2019) and shows that medical mental health professionals may have a negative belief that people with mental illness cannot and do not want to work. The results also support literature showing how some mental health professionals may consider that work may hinder the recovery of people with mental illness (Casper & Carloni, 2007; Hamilton et al., 2013; Marwaha et al., 2009; Mueser & McGurk, 2014) since both psychiatrists and nurses give importance to frustration originated from work. One of the principles of the Individual Placement and Support (IPS) program is that employment services are integrated with mental health treatment services, and IPS is a specific supported employment program for people with mental illness that showed extensive evidence of improved work outcomes when compared to traditional employment rehabilitation services (Bond, 2004; Bond et al., 2020; Kinoshita et al., 2013; Suijkerbuijk et al., 2017). When medical mental health professionals are involved in the process of restoring a person's ability to work, their attitude toward the employability of people with psychiatric disabilities can improve. In this situation, individuals with mental illness can be observed not only while they are in an acute or in a relapsed phase of illness but also while they are engaging in a positive work experience (Corker et al., 2018; Gladman et al., 2015; Hatfield et al., 1992). Consequently, we consider that closer coordination between social and medical mental health organizations is necessary to avoid indirect stigmatizing effects that the attitudes of mental health employees may have on people with psychiatric disabilities (Arvaniti et al., 2009; Lagunes‐Cordoba et al., 2020; Marwaha et al., 2009; Schulze, 2007).

Despite evidence from supported employment programs, such as IPS, where better employment outcomes are achieved for people with psychiatric disabilities (Gewurtz et al., 2012; Grove, 2015; Koletsi et al., 2009; Suijkerbuijk et al., 2017), and given efforts to extend these services in Europe (Beyer et al., 2010), statistics show that people with mental illness remain marginalized from competitive employment (OECD, 2012, 2018). Attitudes of mental health professionals toward the employability of these people may be acting as a barrier, limiting the implementation of inclusive employment services, or diminishing the effectiveness of active work rehabilitation programs.

### 4.1 Practical implications

The results of this study will serve as a reference for specific educational projects to be used in the future that may be developed for professional groups involved in the employment rehabilitation of people with mental illness. Additionally, they can also be used for improving the attitudes of mental health professionals involved in the recovery process of people with psychiatric disabilities.

### 4.2 Limitations

This study should be taken with care and be considered an initial report supporting the future direction of this research. We need to extend the sample size, and despite the previous research analyzing this issue and supporting the conclusions of this work, we cannot generalize these findings. Another important limitation is that we have not collected data about professionals' commitment to supporting people with mental illness for employment activity. Mental health medical professionals typically observe people with mental illness while they are in a passive role as patients receiving psychiatric clinical services. However, in further research, it will be important to quantify professionals’ exposure to contact experiences while people with mental illness are at work or in other occupational activities. We need to corroborate that contact experiences while people with mental illness are active in occupational activities may have a significant association with professionals' attitudes.

## CONFLICT OF INTEREST

All authors have no conflict of interest and no financial support.

### 1 PEER REVIEW

The peer review history for this article is available at: https://publons.com/publon/10.1002/brb3.2767.

## Data Availability

The data that support the findings of this study are available upon reasonable request from the corresponding author.

## References

[brb32767-bib-0002] Acta Sanitaria . (2016). Janssen destaca “el papel clave del enfermero” en el abordaje de la enfermedad mental grave en una jornada celebrada en Zamora. Barbizon S.L . https://www.actasanitaria.com/industria‐farmaceutica/janssen‐destaca‐el‐papel‐clave‐del‐enfermero‐en‐el‐abordaje‐de‐la‐enfermedad‐mental‐grave_1213316_102.html

[brb32767-bib-0003] Arvaniti, A. , Samakouri, M. , Kalamara, E. , Bochtsou, V. , Bikos, C. , & Livaditis, M. (2009). Health service staff's attitudes towards patients with mental illness. Social Psychiatry and Psychiatric Epidemiology, 44(8), 658–665. 10.1007/s00127-008-0481-3 19082905

[brb32767-bib-0004] Asparouhov, T. , & Muthén, B. (2010). Simple second order chi‐square correction . Mplus technical appendix. https://www.statmodel.com/download/WLSMV_new_chi21.pdf

[brb32767-bib-0005] Becker, D. R. , & Drake, R. E. (1994). Individual placement and support: A community mental health center approach to vocational rehabilitation. Community Mental Health Journal, 30(2), 193–206. 10.1007/BF02188630 8013215

[brb32767-bib-0006] Bentler, P. M. (1990). Comparative fit indexes in structural models. Psychological Bulletin, 107(2), 238–246. 10.1037/0033-2909.107.2.238 2320703

[brb32767-bib-0007] Bertilsson, M. , Löve, J. , Ahlborg, G. , & Hensing, G. (2015). Health care professionals’ experience‐based understanding of individuals’ capacity to work while depressed and anxious. Scandinavian Journal of Occupational Therapy, 22(2), 126–136. 10.3109/11038128.2014.985607 25581065

[brb32767-bib-0008] Beyer, S. , Jordán de Urríes, F. d. B. , & Verdugo, M. (2010). A comparative study of the situation of supported employment in Europe. Journal of Policy and Practice in Intellectual Disabilities, 7, 130–136. 10.1111/j.1741-1130.2010.00255.x

[brb32767-bib-0009] Bond, G. R. (2004). Supported employment: Evidence for an evidence‐based practice. Psychiatric Rehabilitation Journal, 27(4), 345–359. 10.2975/27.2004.345.359 15222147

[brb32767-bib-0010] Bond, G. R. , Drake, R. E. , & Becker, D. R. (2020). An update on Individual Placement and Support. World psychiatry: Official journal of the World Psychiatric Association (WPA), 19(3), 390–391. 10.1002/wps.20784 32931093 PMC7491619

[brb32767-bib-0011] Brantschen, E. , Landolt, K. , Kawohl, W. , Rössler, W. , Bärtsch, B. , & Nordt, C. (2017). Two types of expectancies concerning competitive employment among people with mental illness in supported employment. Journal of Vocational Rehabilitation, 46(2), 195–202. 10.3233/JVR-160855

[brb32767-bib-0012] Browne, M. W. , & Cudeck, R. (1992). Alternative ways of assessing model fit. Sociological Methods & Research, 21(2), 230. 10.1177/0049124192021002005

[brb32767-bib-0013] Brucker, D. L. , & Doty, M. (2019). Community mental health center staff attitudes about employment for persons with serious mental illness. Psychiatric Rehabilitation Journal, 42(1), 32–40. 10.1037/prj0000326 30299122

[brb32767-bib-0014] Casper, E. S. , & Carloni, C. (2007). Assessing the underutilization of supported employment services. Psychiatric Rehabilitation Journal, 30(3), 182–188. 10.2975/30.3.2007.182.188 17269268

[brb32767-bib-0015] Cohen, P. , & Cohen, J. (1984). The clinician's illusion. Archives of General Psychiatry, 41(12), 1178–1182. 10.1001/archpsyc.1984.01790230064010 6334503

[brb32767-bib-0016] Corker, E. , Hamilton, S. , Henderson, C. , Weeks, C. , Pinfold, V. , Rose, D. , Williams, P. , Flach, C. , Gill, V. , Lewis‐Holmes, E. , & Thornicroft, G. (2018). Experiences of discrimination among people using mental health services in England 2008–2011. British Journal of Psychiatry, 202(s55), s58–s63. 10.1192/bjp.bp.112.112912 23553696

[brb32767-bib-0017] Corrigan, P. W. , Powell, K. J. , & Rüsch, N. (2012). How does stigma affect work in people with serious mental illnesses? Psychiatric Rehabilitation Journal, 35(5), 381–384. 10.1037/h0094497 23116379

[brb32767-bib-0018] Costa, M. , Baker, M. , Davidson, L. , Giard, J. , Guillorn, L. , Ibáñez, A. G. , Weitz, D. , & O'Connell, M. (2017). Provider perspectives on employment for people with serious mental illness. International Journal of Social Psychiatry, 63(7), 632–640. 10.1177/0020764017725769 28797214

[brb32767-bib-0019] Couture, S. , & Penn, D. (2003). Interpersonal contact and the stigma of mental illness: A review of the literature. Journal of Mental Health, 12(3), 291. 10.1080/09638231000118276

[brb32767-bib-0020] Dolce, J. N. , & Bates, F. M. (2019). Hiring and employing individuals with psychiatric disabilities: Focus groups with human resource professionals. Journal of Vocational Rehabilitation, 50(1), 85–93. 10.3233/JVR-180990

[brb32767-bib-0021] Fleming, C. , Curtis, R. , Martin, E. D. , Kraska, M. , Shippen, M. , & Varda, K. (2019). Perceptions and practices of mental health professionals regarding the employment of people with serious mental illness. Journal of Vocational Rehabilitation, 50(1), 39–48. 10.3233/JVR-180986

[brb32767-bib-0022] Foster, A. L. , Chen, R. K. , Reed, B. J. , Miller, E. , & Carlson, R. (2019). Exploring the effectiveness of a prevocational seminar on self‐efficacy and work motivation among adults residing in an inpatient mental health facility. Journal of Rehabilitation, 85(2), 4–12.

[brb32767-bib-0023] Gewurtz, R. E. , Cott, C. , Rush, B. , & Kirsh, B. (2012). The shift to rapid job placement for people living with mental illness: An analysis of consequences. Psychiatric Rehabilitation Journal, 35(6), 428–434. 10.1037/h0094575 23276235

[brb32767-bib-0024] Gladman, B. , Waghorn, G. , Wishart, L. , & Dias, S. (2015). Reliability of health professionals' perceptions of employment for people with severe mental illness. Journal of Rehabilitation, 81, 3–8.

[brb32767-bib-0025] Grove, B. (2015). International employment schemes for people with mental health problems. BJPsych International, 12(4), 97–99. 10.1192/S2056474000000672 29093874 PMC5618873

[brb32767-bib-0026] Hamilton, A. B. , Cohen, A. N. , Glover, D. L. , Whelan, F. , Chemerinski, E. , McNagny, K. P. , Mullins, D. , Reist, C. , Schubert, M. , & Young, A. S. (2013). Implementation of evidence‐based employment services in specialty mental health. Health Services Research, 48, 2224–2244. 10.1111/1475-6773.12115 24138608 PMC4097836

[brb32767-bib-0027] Hand, C. , & Tryssenaar, J. (2006). Small business employers' views on hiring individuals with mental illness. Psychiatric Rehabilitation Journal, 29(3), 166–173. 10.2975/29.2006.166.173 16450927

[brb32767-bib-0028] Harris, L. M. , Matthews, L. R. , Penrose‐Wall, J. , Alam, A. , & Jaworski, A. (2014). Perspectives on barriers to employment for job seekers with mental illness and additional substance‐use problems. Health & Social Care in the Community, 22(1), 67–77. 10.1111/hsc.12062 23829791

[brb32767-bib-0029] Hatfield, B. , Huxley, P. , & Mohamad, H. (1992). Accommodation and employment: A survey into the circumstances and expressed needs of users of mental health services in a northern town. The British Journal of Social Work, 22(1), 61–73. http://www.jstor.org/stable/23709226

[brb32767-bib-0030] Hillborg, H. , Danermark, B. , & Svensson, T. (2013). Professionals’ perceptions of and views about vocational rehabilitation for people with psychiatric disabilities. Work: Journal of Prevention, Assessment & Rehabilitation, 44(4), 471–480. 10.3233/WOR-131518 23324725

[brb32767-bib-0031] Hong, P. Y. , Polanin, J. R. , Key, W. , & Choi, S. (2014). Development of the Perceived Employment Barrier Scale (PEBS): Measuring psychological self‐sufficiency. Journal of Community Psychology, 42(6), 689–706. 10.1002/jcop.21646

[brb32767-bib-0032] Hu, L. ‐T. , & Bentler, P. M. (1998). Fit indices in covariance structure modeling: Sensitivity to underparameterized model misspecification. Psychological Methods, 3(4), 424–453. 10.1037/1082-989X.3.4.424

[brb32767-bib-0033] Jansson, I. , & Gunnarsson, A. B. (2018). Employers’ views of the impact of mental health problems on the ability to work. Work: Journal of Prevention, Assessment & Rehabilitation, 59(4), 585–598. 10.3233/WOR-182700 29733044

[brb32767-bib-0034] Kinoshita, Y. , Furukawa, T. A. , Kinoshita, K. , Honyashiki, M. , Omori, I. M. , Marshall, M. , Bond, G. R. , Huxley, P. , Amano, N. , & Kingdon, D. (2013). Supported employment for adults with severe mental illness. The Cochrane Database of Systematic Reviews, 2013(9), CD008297. 10.1002/14651858.CD008297.pub2 PMC7433300 24030739

[brb32767-bib-0035] Koletsi, M. , Niersman, A. , van Busschbach, J. T. , Catty, J. , Becker, T. , Burns, T. , Fioritti, A. , Kalkan, R. , Lauber, C. , Rössler, W. , Tomov, T. , & Wiersma, D. (2009). Working with mental health problems: Clients’ experiences of IPS, vocational rehabilitation and employment. Social Psychiatry and Psychiatric Epidemiology, 44(11), 961–970. 10.1007/s00127-009-0017-5 19280083

[brb32767-bib-0036] Lagunes‐Cordoba, E. , Davalos, A. , Fresan‐Orellana, A. , Jarrett, M. , Gonzalez‐Olvera, J. , Thornicroft, G. , & Henderson, C. (2020). Mental health service users’ perceptions of stigma, from the general population and from mental health professionals in Mexico: A qualitative study. Community Mental Health Journal, 57(5), 985–993. 10.1007/s10597-020-00706-4 32892303 PMC8131298

[brb32767-bib-0037] Lettieri, A. , & Díez, E. (2017). A systematization of the international evidence related to labor inclusion barriers and facilitators for people with mental illness. A review of reviews. Sociologica, 3, 5–5. 10.2383/89515

[brb32767-bib-0038] Lettieri, A. , Díez, E. , & Soto‐Pérez, F. (2021). Prejudice and work discrimination: Exploring the effects of employers’ work experience, contact and attitudes on the intention to hire people with mental illness. The Social Science Journal, Advance online publication. 10.1080/03623319.2021.1954464

[brb32767-bib-0039] Lettieri, A. , Díez, E. , Soto‐Pérez, F. , & Bernate‐Navarro, M. (2022). Employment related barriers and facilitators for people with psychiatric disabilities in Spain. Work, 71(4), 901–915. 10.3233/WOR-213642 35253720

[brb32767-bib-0040] Lettieri, A. , Soto‐Pérez, F. , Franco‐Martín, M. A. , Jordán de Urríes, F. d. B. , Shiells, K. R. , & Díez, E. (2021). Employability with mental illness: The perspectives of employers and mental health workers. Rehabilitation Counseling Bulletin, 64(4), 195–207. 10.1177/0034355220922607

[brb32767-bib-0041] Lorenzo‐Seva, U. , & Ferrando, P. J. (2006). FACTOR: A computer program to fit the exploratory factor analysis model. Behavior Research Methods, 38(1), 88–91. 10.3758/BF03192753 16817517

[brb32767-bib-0042] Lorenzo‐Seva, U. , & Ferrando, P. J. (2013). Factor 92: A comprehensive program for fitting exploratory and semiconfirmatory factor analysis and IRT models. Applied Psychological Measurement, 37(6), 497–498. 10.1177/0146621613487794

[brb32767-bib-0043] Mangili, E. , Ponteri, M. , Buizza, C. , & Rossi, G. (2011). Attitudes toward disabilities and mental illness in work settings: A review. Epidemiologia e Psichiatria Sociale, 13(1), 29–46. 10.1017/S1121189/00003213 15248392

[brb32767-bib-0044] Marwaha, S. , Balachandra, S. , & Johnson, S. (2009). Clinicians' attitudes to the employment of people with psychosis. Social Psychiatry and Psychiatric Epidemiology, 44(5), 349–360. 10.1007/s00127-008-0447-5 18979055

[brb32767-bib-0045] Maunder, R. , & White, F. (2019). Intergroup contact and mental health stigma: A comparative effectiveness meta‐analysis. Clinical Psychology Review, 72, 101749. 10.1016/j.cpr.2019.101749 31254936

[brb32767-bib-0046] McGurk, S. R. , & Mueser, K. T. (2013). Cognition and work functioning in schizophrenia. In P. D. Harvey (Ed.), Cognitive impairment in schizophrenia: Characteristics, assessment and treatment (pp. 98–109). Cambridge University Press. 10.1017/CBO9781139003872.007

[brb32767-bib-0047] Moody, L. , Saunders, J. , Leber, M. , Wójcik‐Augustyniak, M. , Szajczyk, M. , & Rebernik, N. (2017). An exploratory study of barriers to inclusion in the European workplace. Disability & Rehabilitation, 39(20), 2047–2054. 10.1080/09638288.2016.1217072 27665671

[brb32767-bib-0048] Morgan, A. J. , Reavley, N. J. , Jorm, A. F. , & Beatson, R. (2016). Experiences of discrimination and positive treatment from health professionals: A national survey of adults with mental health problems. Australian & New Zealand Journal of Psychiatry, 50(8), 754–762. 10.1177/0004867416655605 27354100

[brb32767-bib-0049] Morin, A. J. S. , Arens, A. K. , & Marsh, H. W. (2016). A bifactor exploratory structural equation modeling framework for the identification of distinct sources of construct‐relevant psychometric multidimensionality. Structural Equation Modeling, 23(1), 116–139. 10.1080/10705511.2014.961800

[brb32767-bib-0050] Mueser, K. T. , & McGurk, S. R. (2014). Supported employment for persons with serious mental illness: Current status and future directions. L'Encéphale, 40, S45–S56. 10.1016/j.encep.2014.04.008 24929974

[brb32767-bib-0051] Netto, J. A. , Yeung, P. , Cocks, E. , & McNamara, B. (2016). Facilitators and barriers to employment for people with mental illness: A qualitative study. Journal of Vocational Rehabilitation, 44(1), 61–72. 10.3233/JVR-150780

[brb32767-bib-0052] Noel, V. A. , Bond, G. R. , Drake, R. E. , Becker, D. R. , McHugo, G. J. , Swanson, S. J. , Luciano, A. E. , & Greene, M. A. (2017). Barriers and facilitators to sustainment of an evidence‐based supported employment program. Administration and Policy in Mental Health and Mental Health Services Research, 44(3), 331–338. https://10.1007/s10488‐016‐0778‐6 27891567 10.1007/s10488-016-0778-6

[brb32767-bib-0053] Nordt, C. , Rössler, W. , & Lauber, C. (2006). Attitudes of mental health professionals toward people with schizophrenia and major depression. Schizophrenia Bulletin, 32(4), 709–714. 10.1093/schbul/sbj065 16510695 PMC2632277

[brb32767-bib-0054] Olmo‐Romero, F. , González‐Blanco, M. , Sarró, S. , Grácio, J. , Martín‐Carrasco, M. , Martinez‐Cabezón, A. C. , Perna, G. , Pomarol‐Clotet, E. , Varandas, P. , Ballesteros‐Rodríguez, J. , Rebolleda‐Gil, C. , Vanni, G. , & González‐Fraile, E. (2019). Mental health professionals’ attitudes towards mental illness: Professional and cultural factors in the INTER NOS study. European Archives of Psychiatry and Clinical Neuroscience, 269(3), 325–339. 10.1007/s00406-018-0867-5 29353369

[brb32767-bib-0001] Ottewell, N. (2019). The association between employers' mental health literacy and attitudes towards hiring people with mental illness. Work, 64(4), 743–753. 10.3233/WOR-193036 31815714

[brb32767-bib-0055] Organisation for Economic Co‐operation and Development . (2012). Sick on the Job? Myths and realities about mental health and work. OECD Publishing. 10.1787/9789264124523-en

[brb32767-bib-0056] Organisation for Economic Co‐operation and Development . (2018). Recommendation of the council—Integrated mental health, skills and work policy. OECD. http://www.oecd.org/els/emp/Flyer_MHW%20Council%20Recommendation.pdf

[brb32767-bib-0057] Ozawa, A. , & Yaeda, J. (2007). Employer attitudes toward employing persons with psychiatric disability in Japan. Journal of Vocational Rehabilitation, 26(2), 105–113. https://content.iospress.com/articles/journal‐of‐vocational‐rehabilitation/jvr00369

[brb32767-bib-0058] Porter, S. , Lexén, A. , & Bejerholm, U. (2019). Mental health literacy among vocational rehabilitation professionals and their perception of employers in the return‐to‐work process. Journal of Vocational Rehabilitation, 50(2), 157–169. 10.3233/JVR-180997

[brb32767-bib-0059] Porter, S. , Lexén, A. , Johanson, S. , & Bejerholm, U. (2018). Critical factors for the return‐to‐work process among people with affective disorders: Voices from two vocational approaches. Work, 60, 221–234. 10.3233/WOR-182737 29843300 PMC6027949

[brb32767-bib-0060] Reddy, L. F. , Llerena, K. , & Kern, R. S. (2016). Predictors of employment in schizophrenia: The importance of intrinsic and extrinsic motivation. Schizophrenia Research, 176(2‐3), 462–466. 10.1016/j.schres.2016.08.006 27567733

[brb32767-bib-0061] Rinaldi, M. , Perkins, R. , Glynn, E. , Montibeller, T. , Clenaghan, M. , & Rutherford, J. (2008). Individual placement and support: From research to practice. Advances in Psychiatric Treatment, 14(1), 50–60. 10.1192/apt.bp.107.003509

[brb32767-bib-0062] Roets, G. , Kristiansen, K. , Van Hove, G. , & Vanderplasschen, W. (2007). Living through exposure to toxic psychiatric orthodoxies: Exploring narratives of people with ‘mental health problems’ who are looking for employment on the open labour market. Disability & Society, 22(3), 267–281. 10.1080/09687590701259559

[brb32767-bib-0063] Rose, G. , von Hippel, C. , Brener, L. , & von Hippel, B. (2018). Attitudes of people working in mental health non‐governmental organisations in Australia: A comparison with other mental health professionals. Health Psychology Open, 5(1), 2055102918765413. 10.1177/2055102918765413 29721330 PMC5922491

[brb32767-bib-0064] Rüsch, N. , Nordt, C. , Kawohl, W. , Brantschen, E. , Bärtsch, B. , Müller, M. , Corrigan, P. W. , & Rössler, W. (2014). Work‐related discrimination and change in self‐stigma among people with mental illness during supported employment. Psychiatric Services, 65(12), 1496–1498. 10.1176/appi.ps.201400073 25270160

[brb32767-bib-0065] Ryan, P. , Ramon, S. , & Greacen, T. (2012). Empowerment, lifelong learning and recovery in mental health: Towards a new paradigm. Palgrave Macmillan/Springer Nature. 10.1007/978-0-230-39135-2

[brb32767-bib-0066] Saavedra, J. , López, M. , Gonzáles, S. , & Cubero, R. (2016). Does employment promote recovery? Meanings from work experience in people diagnosed with serious mental illness. Culture, Medicine, and Psychiatry: An International Journal of Cross‐Cultural Health Research, 40(3), 507–532. 10.1007/s11013-015-9481-4 26581838

[brb32767-bib-0067] Schulze, B. (2007). Stigma and mental health professionals: A review of the evidence on an intricate relationship. International Review of Psychiatry, 19(2), 137–155. 10.1080/09540260701278929 17464792

[brb32767-bib-0068] Steiger, J. H. (1990). Structural model evaluation and modification: An interval estimation approach. Multivariate Behavioral Research, 25(2), 173–180. 10.1207/s15327906mbr2502_4 26794479

[brb32767-bib-0069] Suijkerbuijk, Y. B. , Schaafsma, F. G. , van Mechelen, J. C. , Ojajärvi, A. , Corbiere, M. , & Anema, J. R. (2017). Interventions for obtaining and maintaining employment in adults with severe mental illness, a network meta‐analysis. Cochrane Database of Systematic Reviews, 9(9), CD011867. 10.1002/14651858.CD011867.pub2 28898402 PMC6483771

[brb32767-bib-0070] Torres Stone, R. A. , Sabella, K. , Lidz, C. W. , McKay, C. , & Smith, L. M. (2018). The meaning of work for young adults diagnosed with serious mental health conditions. Psychiatric Rehabilitation Journal, 41(4), 290–298. 10.1037/prj0000195 27295134

[brb32767-bib-0071] Tucker, L. R. , & Lewis, C. (1973). A reliability coefficient for maximum likelihood factor analysis. Psychometrika, 38(1), 1–10. 10.1007/BF02291170

[brb32767-bib-0072] Unger, D. D. (2002). Employers' attitudes toward persons with disabilities in the workforce: Myths or realities? Focus on Autism and Other Developmental Disabilities, 17(1), 2–10. 10.1177/108835760201700101

[brb32767-bib-0073] Waghorn, G. , Lockett, H. , & King, J. (2020). Practices of higher performing employment specialists when helping job‐seekers with psychiatric disabilities to obtain competitive employment. Journal of Rehabilitation, 86(2), 22–30.

[brb32767-bib-0074] Waghorn, G. , Logan, J. , Dias, S. , & Hielscher, E. (2017). Vocational functioning among people with psychiatric disabilities five to seven years after receiving supported employment services. Journal of Rehabilitation, 83(2), 36–42.

[brb32767-bib-0075] World Health Organization (2000). Mental health and work: Impact, issues and good practices. WHO. http://www.who.int/mental_health/media/en/712.pdf

[brb32767-bib-0076] World Health Organization . (2001). International classification of functioning, disability and health (ICF). WHO. http://apps.who.int/iris/bitstream/handle/10665/42407/9241545429.pdf;jsessionid=FA97F44560D24F61A04BDECB9A4F8824?sequence=1

